# Effectiveness and safety of KunXian capsule for the treatment of IgA nephropathy

**DOI:** 10.1186/s12882-022-02814-7

**Published:** 2022-05-10

**Authors:** Wei-Bo Le, Jin-Song Shi, Si-Wen Gong, Fan Yang

**Affiliations:** 1 National Clinical Research Center of Kidney Diseases, Jinling Hospital, Nanjing University School of Medicine, Nanjing, 210002 China; 2 National Clinical Research Center of Kidney Diseases, Jinling Hospital, Nanjing University School of Medicine, Nanjing, 210018 China; 3 National Clinical Research Center of Kidney Diseases, Jinling Hospital, The First School of Clinical Medicine, Southern Medical University, Nanjing, China

**Keywords:** IgA nephropathy, Tripterygium Wilfordii hook F, KunXian, Glomerular disease, Proteinuria, Oligomenorrhea

## Abstract

**Background:**

Tripterygium Wilfordii Hook F (TwHF) preparation has been widely used in the treatments of IgA nephropathy (IgAN) in China. However, the effectiveness and safety of the new generation of TwHF preparation, KuxXian capsule, on the treatment of IgAN remains unknown.

**Methods:**

Here, we retrospectively describe our experience treating 55 consecutive IgAN patients with KunXian. We defined complete remission as proteinuria < 0.5 g/24 h and partial remission as proteinuria < 1 g/24 h, each also having > 50% reduction in proteinuria from baseline.

**Results:**

At first follow-up after KunXian treatment (5.7 weeks, IQR 4.7–7.9), all but two patients (96%) showed a reduction in proteinuria. The overall median proteinuria decreased from 2.23 g/day at baseline to 0.94 g/day (*P* < 0.001) at the first follow-up. During a median follow-up of 28 weeks after KunXian administration, 25(45.5%) patients achieved complete remission, 34 (61.8%) patients achieved complete/partial remission. Of the 12 patients discontinued KunXian treatment during the follow-up, the median proteinuria was increased from 0.97 g/24 h to 2.74 g/24 h after a median of 10.9 weeks (*P* = 0.004). Multivariable Cox models showed that female, treatment switching from previous generation of TwHF preparation, lower initial KunXian dosage, and higher proteinuria at baseline were independently associated proteinuria remission. Of the 20 pre-menopausal females, 12 of them developed oligomenorrhea or menstrual irregularity and ten of them developed amenorrhea.

**Conclusion:**

KunXian is effectiveness and safety for the treatment of IgA nephropathy. Woman of childbearing age to be informed of the risk of ovarian failure after being treated with TwHF preparations.

## Introduction

IgA nephropathy (IgAN) is the most common cause of primary glomerulonephritis worldwide [1, 2]. Slow progression to end-stage kidney disease occurs in up to 25 to 34% of affected patients within 20 to 25 years of presentation [2, 3]. The optimal approach to the treatment of IgA nephropathy is uncertain. Two current approaches were used for IgAN therapy: optimized supportive care for all patients and immunosuppressive therapy for high-risk patients [4]. There is an alternative special approach, *Tripterygium Wilfordii* Hook F (TwHF) preparation treatment, for patients with IgAN in China. TwHF preparations were extracted from the Chinese herb TwHF (also known as thunder god vine, Lei Gong Teng and Huang Teng), which is a vine plant belonging to the genus Tripterygium of the Celastraceae family and has been used in traditional Chinese medicine for more than five hundred years. TwHF preparations were successfully used to treat lepra reactions since 1962 and rheumatoid arthritis since1969 in China [5]. In the late 1970s, TwHF preparations were used to successfully treat patients with glomerular disease in our kidney center [6, 7]. Ten years later, we published the data of treating IgAN with TwHF preparations and found that 72% of patients showed decreased urinary protein excretion by at least 50% from baseline at 6 months [8].

The primary biologically active ingredient of TwHF, triptolide, has various promising pharmacological activities, such as immunosuppressive, anti-inflammatory, antiproliferative, antifertility, antitumor, antiosteoporosis, and neuroprotective properties [9]. We previously found that triptolide could directly protect podocytes in various glomerular disease models in vivo and in vitro [10–13]. We also published two small randomized controlled clinical trial to treat diabetic nephropathy [14] and membranous nephropathy [15] with TwHF. We found that TwHF can significantly reduce proteinuria in those patients. Currently, TwHF is widely used in China to treat various proteinuric kidney disease [8, 14–20]. In the real world, TwHF preparation has been regarded as one of the most widely used therapies for patients with IgAN in China.

The therapeutic and toxicity effects of TwHF preparation is different according to different extraction and purification procedures. Forty years ago, the first generation of TwHF preparation was the raw traditional Chinese herb which is the plant TwHF decocted with water (extraction by boiling of herbal material) [8]. There was no quality control standard for the first generation of TwHF preparation. The second generation of TwHF preparation, named *Tripterygium Wilfordii* multiglycoside (TW), were commercially available in the early 1980s. TW was extracted and purified from TwHF using organic solvents. Wilforlide A (> 10 μg/tablet) was used as the quality control [21, 22]. TW was purified with organic solvent extraction; thus, the toxicity effects related with organic solvents cannot be totally avoided. As a diterpenoid triepoxide, triptolide, was identified as the primary biologically active ingredient of TwHF [23, 24]. Recently, a new generation of TwHF preparations, named KunXian capsule (KX) produced by the pharmaceutical factory of ChenLiJi, has been commercially available in China. Compared with TW, KX is improved regarding purification and quality control procedures. KX is purified by macroporous adsorption resin column technology [12]. Quantitative analysis of triptolide by HPLC was used as the quality control. KX has been approved and widely used to treat rheumatoid arthritis in China [25]. However, the effectiveness and safety of KX in the treatment of IgAN remains unknown. In this study, we describe our experience treating 55 consecutive IgAN patients with persistent proteinuria with KX.

## Methods

Since October 2018, we started to use KX treatment for consecutive patients referred to our IgAN outpatient unit of the National Clinical Research Center of Kidney Diseases. Sixty-three biopsy-proven IgAN patients were treated with KX between October 2018 and October 2020. The standard dosage of KX is six capsules per day (150 μg /d) divided into two or three doses [26, 27]. The initial dosage of KX was 3–6 capsules per day (75–150 μg /d) divided into two or three doses. The maximum dose was six capsules per day. All patients were told to take KX capsules with food to reduce stomach irritation. After enrollment, previous immunosuppressants were stopped or tapered.

The estimated glomerular filtration rate (eGFR) was estimated using the XiangYa equation [28]. The proteinuria measurement was the 24-hour urine collection, based on a chemical assay (biuret reaction). We defined complete remission as proteinuria < 0.5 g/24 h and partial remission as proteinuria < 1 g/24 h, each also having > 50% reduction in proteinuria from baseline. Patients not fulfilling the above criteria were considered as non-remissions. Liver injury was defined as elevated alanine aminotransferase (ALT) or aspartate aminotransferase (AST) levels more than two-fold of the baseline and two-fold of the upper limit of normal (ULN, > 100 U/L).

### Statistical analyses

The Kaplan – Meier method was used to plot the probability of achieving complete remission, complete/partial. Survival time was determined from the beginning of the first KX treatment until the event of interest. Patients not achieving remission were considered as censored at the time of the last visit. In the complete/partial remission composite endpoint the survival time for participants was time to event until complete/partial remission. In a multivariable context, the Cox regression model was carried out to test predictors of achieving complete remission and complete/partial remission composite endpoint. Due to its skewed distribution, proteinuria was square root-transformed before statistical analyses.

The data of baseline and follow-up characteristics were presented as numbers and percentages, means and SDs, or medians and IQRs, as appropriate. Paired t-test, Wilcoxon signed-rank test (paired samples Wilcoxon test), or paired Kruskal-Wallis test was used to compare the differences between baseline and follow-up. Categorical variables were compared using the Pearson χ^2^ test or Fisher’s exact test. All statistical analyses were carried out using R (version 4.0.5). Two-tailed *P* < 0.05 findings were considered statistically significant.

## Results

By October 2020, 63 consecutive patients with IgAN were treated with KX in the Outpatient Clinic of the IgA Nephrology Unit (Table 1). Eight patients were excluded from the analysis: six for proteinuria < 1.0 g/d at baseline, one for stopping KX after 12 days of treatment because for heart palpitations, and one for no follow-up data within 3 months of treatment. The patient who stopped treatment due to heart palpitations had a previous history of myocardial infarction. Finally, a total of 55 patients were available for statistical analyses. At baseline, five (9.1%) patients were not receiving RASi; one patient could not tolerate RASi treatment for low blood pressure; four patients received other anti-hypertension medications for significantly low eGFR or previous hyperkalemia. After the initialization of KX treatment, these five patients were still not treated with RASi during follow-up. Forty-seven patients (85.5%) had been previously treated with immunosuppressants, including TW (33 cases), leflunomide (22 cases), glucocorticoid (13 cases), mycophenolate mofetil (7 cases), FK506 (1 case), cyclophosphamide (1 case), and hydroxychloroquine (1 case). Thirty-six (65.5%) patients were still receiving immunosuppressants at baseline, including TW (20 cases), LFM (13 cases), glucocorticoid (7 cases), and mycophenolate mofetil (4 cases).

**Table 1:** Baseline characteristics of patients in the study group

Characteristic	Baseline (*n* = 55)	At First Visit (*n* = 55)	*P*-value
Age (years)			
Female sex, n (%)	24 (43.6%)	–	
Diabetes mellitus	4 (7.3%)		
Previous immunosuppressants	48 (87.3%)	–	
Receiving immunosuppressants at baseline^a^	36 (65.5%)		
Receiving TW at baseline^a^	20 (36.4%)	–	
Receiving RASi at baseline	50 (90.9%)	–	
Proteinuria (g/24 h)	2.2 (1.5–3.2)	0.94 (0.61–1.65)	< 0.001
Mean artery pressure (MAP, mmHg)	94.4 ± 10.6	89.1 ± 10.9	< 0.001
Estimated GFR (ml/min per 1.73 m^2^)	65 ± 13	65 ± 14	0.71
Estimated GFR < 60 ml/min per 1.73 m^2^	21 (38.2%)	21 (38.2%)	
Serum albumin (g/dl)	4.05 ± 0.35	3.66 ± 0.42	< 0.001
Total cholesterol (mg/dL)	216.6 ± 46.4	247.5 ± 50.3	< 0.001
Triglyceride ((mg/dL)	168.3 ± 79.7	141.7 ± 70.9	0.06
LDL cholesterol (mg/dL)	119.9 ± 34.8	147.0 ± 46.4	< 0.001
HDL cholesterol (mg/dl)	46.4 ± 11.6	61.9 ± 15.5	< 0.001
White blood cell count (WBC, 10^9/L)	6.9 ± 2.4	6.1 ± 2.1	0.006
Alanine aminotransferase (ALT, U/L)	24 (14–37)	27 (21–49)	< 0.001
Aspartate aminotransferase (AST, U/L)	23 (20–29)	27 (23–32)	< 0.001

*TW* tripterygium Wilfordii multiglycoside (the second generation of TwHF preparations), *RASi* Renin-angiotensin system inhibitors; ^a^ TW is also considered as an immunosuppressant

There were 20 patients stopped TW and switched to KX therapy after enrollment. There were no differences in the levels of baseline urine protein (2.23 g/24 h, IQR 1.51–2.95 vs. 2.17 g/24 h, IQR 1.66–3.49, *P* = 0.57) or eGFR (66 ± 12 vs. 63 ± 14 ml/min per 1.73m^2^, *P* = 0.30) between patients switched from TW therapy and those who did not. There were 24 women and 31 men in this study. Females had a lower urine protein at baseline than males (1.97 g/24 h, IQR 1.50–2.44 vs 2.37 g/24 h, IQR 1.78, 3.57), while eGFR was similar (66 ± 12 vs. 64 ± 13 ml/min per 1.73m^2^, *P* = 0.51). There were 34 patients who received the standard dose of KX (six capsules per day), 12 received four capsules per day, and nine received three capsules per day.

### Proteinuria changes at the first and second follow-ups after KX treatment

**Fig. 1:**
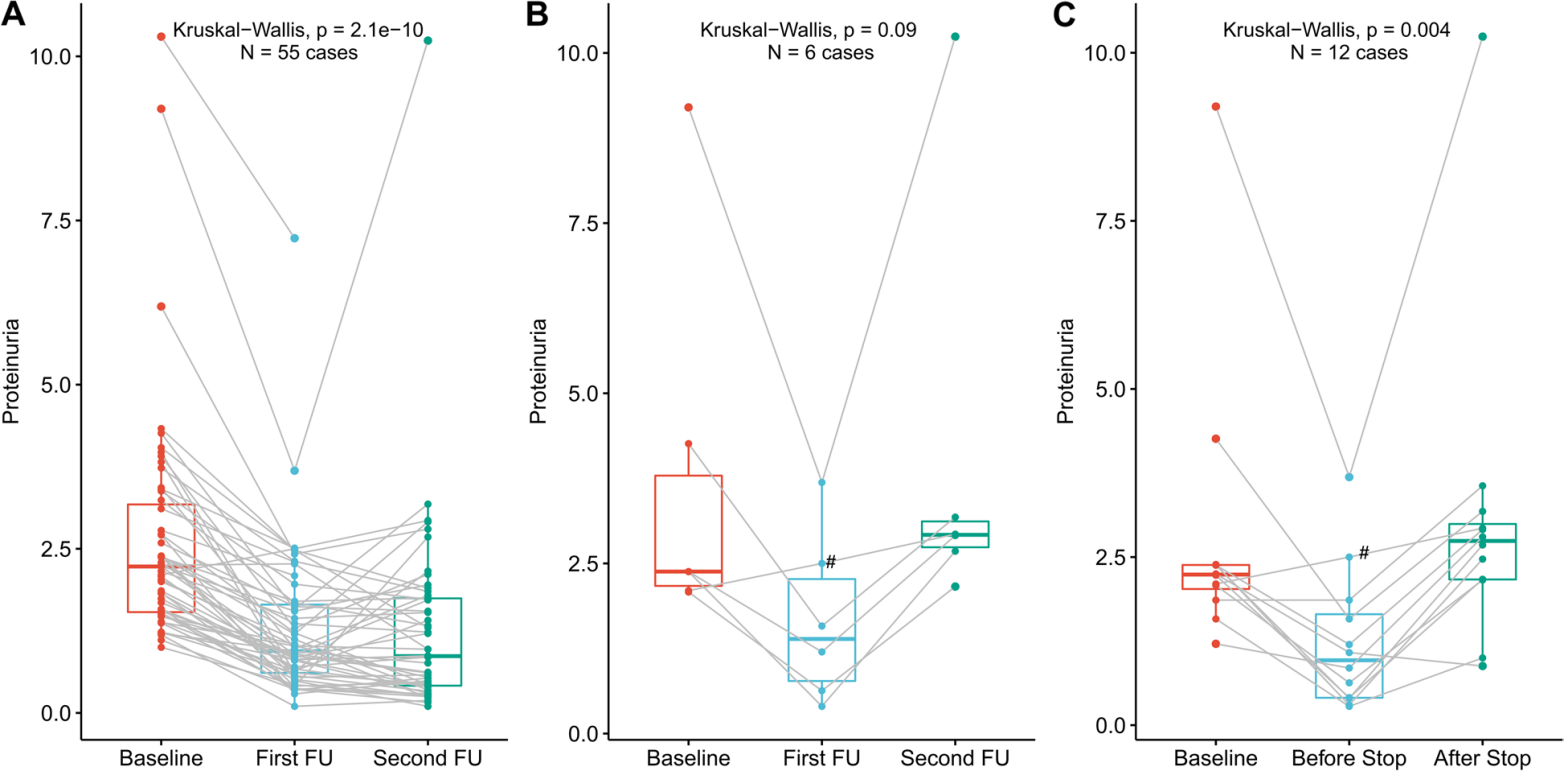
Proteinurias change after KunXian treatment (**A**) and discontinued the treatment (**B** and **C**). Panel **A** shows proteinuria changes from baseline to the first follow-up after KunXian treatment. All but two (96%, green lines) patients showed a reduction in proteinuria at the first follow-up. Panel **B** shows proteinuria changes of the six patients who discontinued the KX treatment at first follow-up visit. Panel **C** shows proteinuria changes of the twelve patients who discontinued the KX treatment during the full follow-up. Eleven of the twelve patients (91.7%) showed an increase in proteinuria after discontinued the KX treatment. # This patient discontinued the KX treatment for no improvement of the disease at the first follow-up visit. FU, Follow-up; KX, KunXian

Figure 1A shows the proteinuria changes from baseline to the first follow-up after KX treatment. All but two (96%) patients showed a reduction in proteinuria at the first follow-up (5.7 weeks, IQR 4.7–7.9 weeks). The overall median proteinuria decreased from 2.23 g/day (IQR 1.54–3.18) at baseline to 0.94 g/day (IQR 0.61–1.65, *P* < 0.001) at the first follow-up. The overall percentage in reduction of proteinuria from baseline was 50.4% (IQR 37.8–66.7%). A total of 23 patients (41.8%) achieved complete/partial remission and 10 patients (18.2%) achieved complete remission. The characteristics of patients with complete/partial or no remission at first follow-up were shown in Table 2. There were 27 patients followed up within 5 weeks (4.7, IQR 4.4–4.9) after KX treatment, three (11.1%) of whom achieved complete remission and nine (33.3%) of whom achieved complete/partial remission.

**Table 2:** Characteristics of patients with complete/partial or no remission at First Follow-up

Characteristic	*n* = 32	Remissions *n* = 23	*P*-value
Age (years)	39.5 ± 8.2	36.9 ± 7.7	0.24
Female sex, n (%)	23 (71.9%)	8 (34.8%)	0.01
Diabetes mellitus	4 (12.5%)	0 (0.0%)	0.22
Previous immunosuppressants^a^	28 (87.5%)	20 (87.0%)	1.0
Receiving immunosuppressants at baseline^a^	23 (71.9%)	13 (56.5%)	0.37
Receiving TW at baseline^a^	16 (50.0%)	4 (17.4%)	0.03
Receiving RASi at baseline	27 (84.4%)	23 (100.0%)	0.13
Proteinuria (g/24 h)	2.4 (1.9, 3.5)	1.8 (1.5, 2.3)	0.01
Mean artery pressure at baseline (mmHg)	96.8 ± 11.0	91.0 ± 9.2	0.05
estimated GFR (ml/min per 1.73 m^2^)	63 ± 13	68 ± 11	0.14
eGFR < 60 ml/min per 1.73 m^2^	15 (46.9)	6 (26.1)	0.20
Serum albumin (g/dl)	39.6 (3.5)	41.7 (3.3)	0.03
KuXian Dosage (ug)	102 ± 24	100 ± 26	0.71

*TW* tripterygium Wilfordii multiglycoside (the second generation of TwHF preparations), *RASi* Renin-angiotensin system inhibitors; ^a^ TW is also considered as an immunosuppressant

There were 50 patients available for analysis at the second follow-up after treatment (17.4 weeks, IQR 13.9–20.5 weeks). At the second follow-up, the median proteinuria of the 50 patients was further decreased to 0.87 g/day (IQR 0.43–1.75, Fig. 1B). A total of 25 patients (26 of 50, 52%) achieved complete/partial remission and 16 patients (17 of 50, 34%) achieved complete remission. Among the 50 patients, six patients stopped KX treatment within 3 months and before the second follow-up (Fig. 1B). Three patients stopped KX treatment themselves for financial and economic issues; two patients stopped taking KX as recommended by other doctors for unknown reason; and one patient stopped due to no improvement of the disease. If the six patients who discontinued the treatment were excluded, the median proteinuria was 0. 69 g/day (IQR 0.39–1.44) at second follow-up visit after KX treatment. In addition, 25 patients (26 of 44, 59.1%) achieved complete/partial remission and 16 patients (17 of 44, 38.6%) achieved complete remission at the second follow-up.

### Proteinuria remission after KX treatment

Over a median follow-up of 28 (IQR, 14–60) weeks, 25 (45.5%) patients achieved complete remission, 34 (61.8%) patients achieved complete/partial remission, and 41 patients (74.5%) achieved > 50% reduction in proteinuria from baseline values after KX administration. Partial/complete remission was achieved over a median of 8.4 weeks (IQR, 5.1–13.9), and complete remission was achieved over a median of 14.7 weeks (IQR, 7.7–27).

**Fig. 2:**
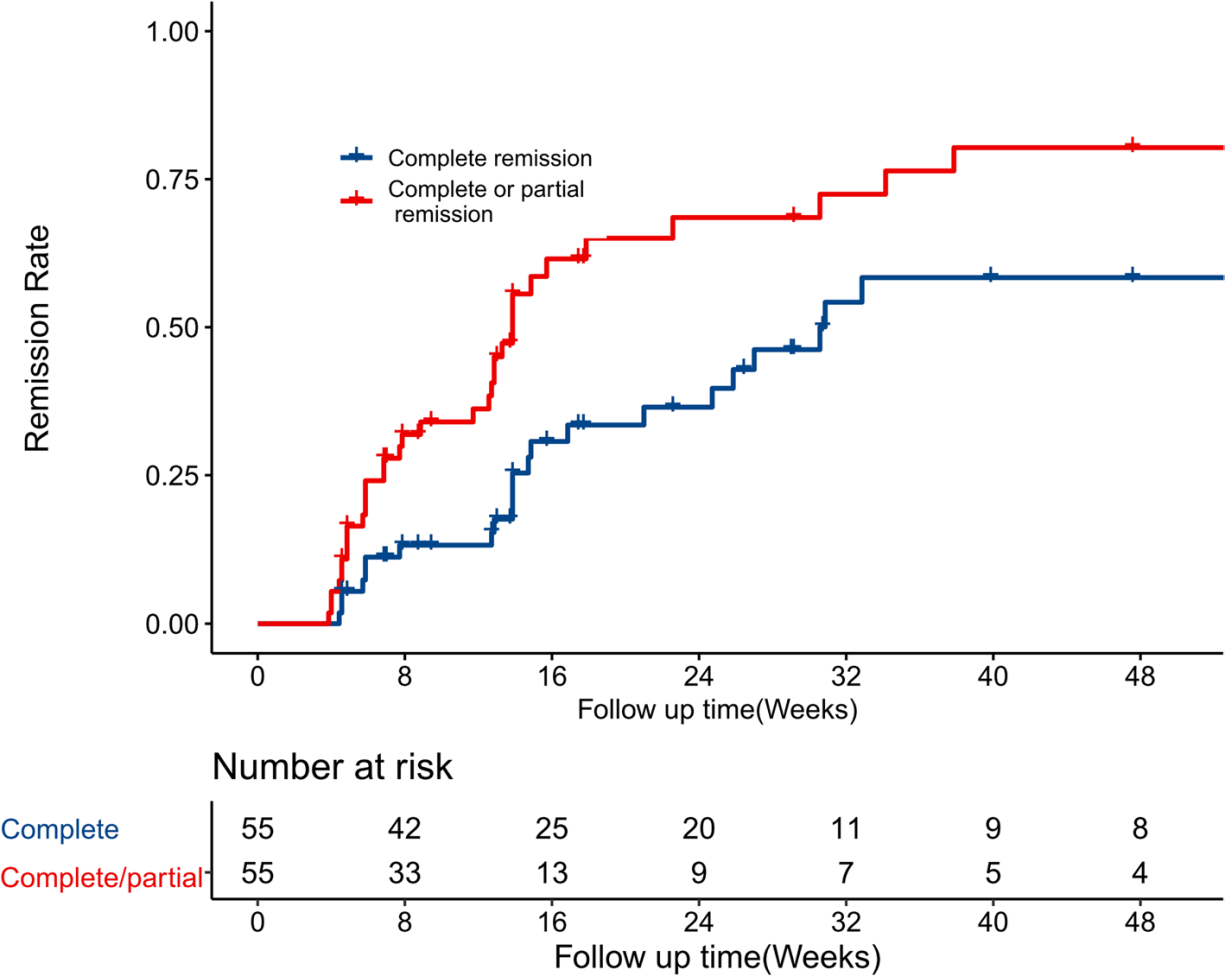
Kaplan–Meier curves of complete remission (blue) and complete/partial remission (red) after KunXian therapy

As shown in Fig. 2, the cumulative complete remission rates at 8-, 16- and 24-weeks were 13% (95% CI, 5–22%), 31% (95% CI, 15–43%) and 40% (95% CI, 22–53%) respectively, and the complete/partial rates were 32% (95% CI, 18–43%), 61% (95% CI, 43–74%) and 68% (95% CI, 49–80%) respectively.

### Proteinuria changes when discontinued the KX treatment

As described previously, six patients discontinued KX treatment before the second follow-up (Fig. 1B). An additional six patients discontinued KX during the follow-up. Two patients discontinued KX due to financial and economic issues, two discontinued KX medication due to it being out of stock locally, and two discontinued due to amenorrhea. Thus, a total of 12 patients discontinued KX treatment during the follow-up. Fig. 1D shows proteinuria changes after discontinuation of the KX treatment for each individual. The median proteinuria was increased from 0.97 g/24 h (IQR 0.41–1.65) immediately to 2.74 g/24 h (IQR 2.17–2.99) in a median of 10.9 weeks (IQR 6.3–22.1 weeks) after discontinuation of the KX treatment (*P* = 0.004).

### Factors associated with proteinuria remission after KX treatment

**Fig. 3:**
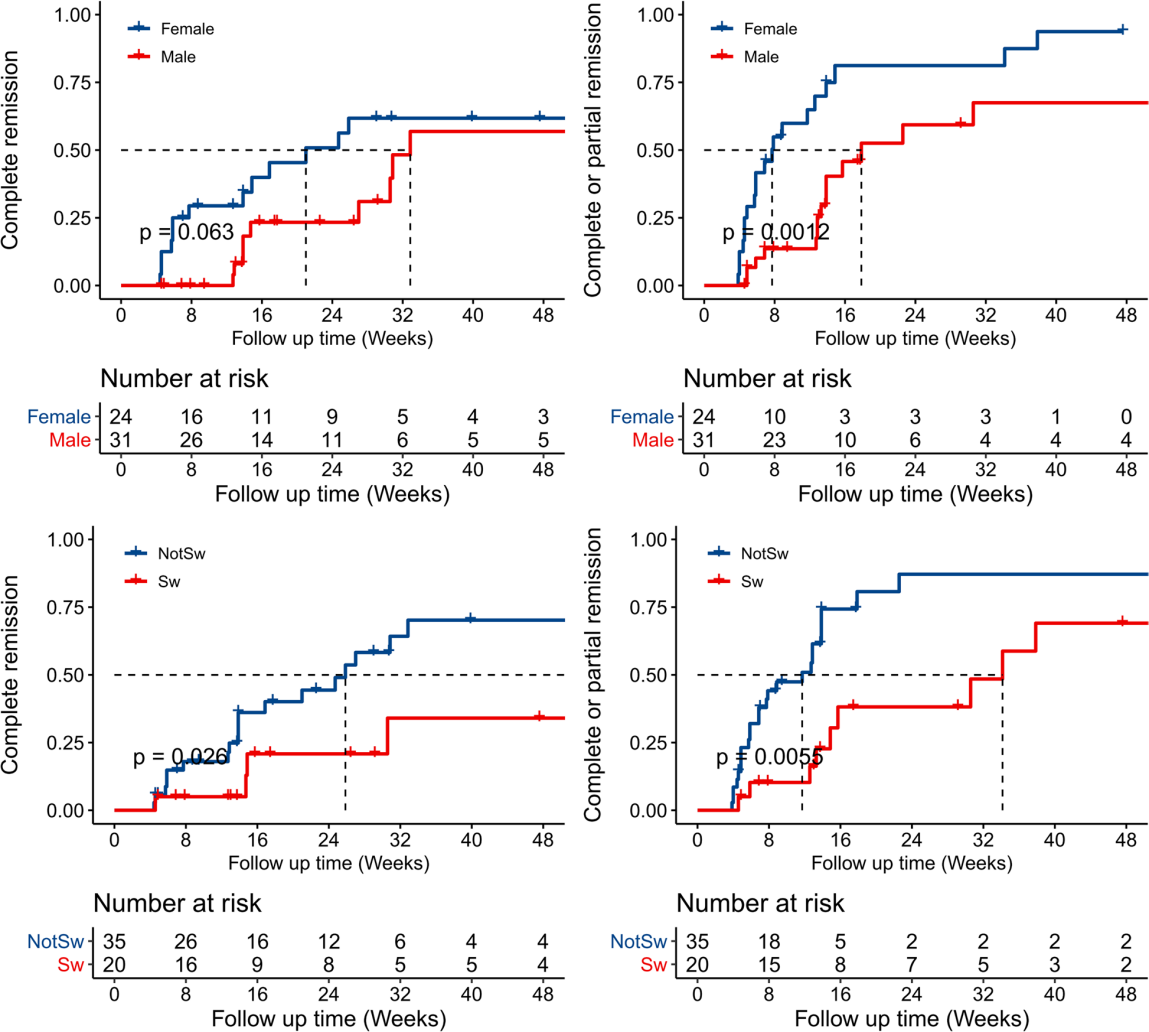
The Kaplan–Meier curves of complete and complete/partial remission according to sex (panel **A** and **B**) and treatment switching (from TW or not from TW, panel **C** and **D**). Sw, patients switching from tripterygium Wilfordii multiglycoside (TW, the second generation of TwHF preparations); NotSw, patients not switching from TW

Multivariable Cox models were used to analyze factors associated with proteinuria remission after KX treatment (Table 3). Females had a higher rate of complete/partial remission (HR 3.67, 95%CI 1.63–8.27, *P* = 0.001) and complete remission (3.62, 95%CI 1.39–9.42, *P* = 0.008) than males. Twenty patients were being treated with TW before starting KX therapy. After enrollment in this study, they were switched to KX therapy. Of the 20 patients, five (25%) achieved complete remission and nine (45%) achieved complete/partial remission during the treatment. Of the 35 patients not switching from TW therapy, 20 patients (57.1%) had complete remission, and 29 (71.4%) had complete/partial remission. The multivariable Cox models showed that patients switching from TW had a significantly lower rate of complete/partial remission (HR 0.22, 95% CI 0.08–0.55, *P* = 0.001) and complete remission (0.15, 95% CI 0.04–0.57, *P* = 0.005) than those switching from other therapies. In this study, patients with higher proteinuria at baseline were more likely to receive a higher dosage of KX treatment. The initial dosage of KX was not associated with proteinuria remission in the univariate Cox models. However, after adjusting for sex, age, treatment switching type, eGFR, mean artery pressure at baseline and proteinuria at baseline, the initial KX dosage was significantly associated with complete remission (HR 2.62, 95% CI 1.47–4.69, *P* = 0.001) and marginally associated with complete/partial remission (HR 1.33, 95% CI 0.93–1.91, *P* = 0.12). The eGFR at baseline was not associated with protein remission, suggesting the effectiveness of KX is not affected by kidney function. The cumulative complete and complete/partial remission according to sex and treatment switching type were shown in Fig. 3.

**Table 3:** Factors associated for Complete/Partial or Complete remission after KunXian treatment in patients with IgA Nephropathy (*n* = 55)

Factor ^a^	Complete or Partial remission			Complete remission		
	HR	95% CI	*P* value	HR	95% CI	*P* value
Sex (Female)	3.67	1.63–8.27	0.001	3.62	1.39–9.42	0.008
Age at baseline (For every 10 years old)	0.98	0.93–1.03	0.41	1.01	0.95–1.07	0.68
Proteinuria at baseline (g/24 h) ^b^	0.17	0.04–0.85	0.03	0.02	0.002–0.29	0.003
eGFR at baseline (For every 10 ml/min per 1.73 m^2^)	0.92	0.66–1.29	0.64	1.49	0.88–2.55	0.14
Mean artery pressure at baseline (For every 10 mmHg)	1.03	0.97–1.55	0.88	0.85	0.49–1.48	0.57
Switching from TW	0.22	0.08–0.55	0.001	0.15	0.04–0.57	0.005
KX Dosage (For every 20μg per capsule)	1.33	0.93–1.91	0.12	2.62	1.47–4.69	0.001

*HR* hazard rate of complete or partial remission, *eGFR* estimated glomerular filtration rate, *TW* tripterygium Wilfordii multiglycoside (the second generation of TwHF preparations), *KX* KunXian capsule

^a^ Multivariable Cox proportional hazards models were used; ^b^ Proteinuria was square root-transformed before statistical analyses

A multivariable linear regression model showed that after adjusting for age, follow-up time, proteinuria, mean artery pressure, and eGFR at baseline, the percentage reduction of proteinuria from baseline to the first follow-up was associated with sex (11.3 ± 6.3% for females compared with males, *P* = 0.08), initial KX dosage (6.4 ± 2.4% for every capsule, *P* = 0.01), and treatment switching type (− 13.0 ± 2.4% for patients switching from TW therapy compared with patients switching from other therapies, *P* = 0.01).

### Safety and adverse events

KX was well tolerated, and adverse events are listed in Table 4. The most obvious adverse event during treatment was amenorrhea for premenopausal females. There were 24 female patients. Four of them were excluded from the analysis of amenorrhea: two were older than 50 years old and two had amenorrhea before the KX treatment. Of the 20 premenopausal females, 12 developed oligomenorrhea or menstrual irregularity, and 10 developed amenorrhea. The median time to develop amenorrhea was 5.3 (IQR 3.5–8.5) months. Notably, of the 12 premenopausal females who developed oligomenorrhea or menstrual irregularity, all achieved complete/partial remission (100%), and nine of them (75%) achieved complete remission during the follow-up. Of the ten patients who developed amenorrhea, all of them achieved complete/partial remission (100%) and eight of them (80%) achieved complete remission.

**Table 4:** Adverse Events Related to KX treatment in the study

Adverse Events	N (%)
Oligomenorrhea or menstrual irregularity ^a^	12 of 20 (60%)
Amenorrhea ^a^	10 of 20 (50%)
Abdominal pain	2
Liver injury^b^	3 (5.5%)
Skin pigmentation	2

Data are shown as counts (%)

^a^ Four females were excluded in the analysis: two patients with age > 50 years old; two patients had amenorrhea or amenorrhea before KX treatment. ^b^ Liver injury, Liver injury was defined as elevated ALT or AST levels more than two-fold of the baseline and two-fold of the upper limit of normal (ULN, > 100 U/L)

One patient occurred liver injury at the first follow-up. Another two cases of liver injury were observed during follow-up. As shown in Table 1, KX treatment was associated with decreased serum albumin level, decreased white blood cell count, increased total cholesterol (216.6 ± 46.4 vs. 247.5 ± 50.3 mg/dL, *P* < 0.001), increased LDL cholesterol, increased HDL cholesterol, slightly increased ALT and slightly increased AST from baseline to the first follow-up. The serum albumin levels were increased to 37.7 ± 0.42 g/dl at the second follow-up (*P* = 0.03, compared with those at the first follow-up).

## Discussion

TwHF preparation to treat proteinuric kidney disease have been used for over 40 years in China [6]. In this study, we confirmed that the novel generation of TwHF preparations, KX, had a powerful ability to reduce proteinuria in patients with IgAN. As mentioned above, 87.3% of patients had been treated previously with immunosuppressants, and 65.5% were still being treated with immunosuppressants at baseline. Thus, most patients enrolled in this study were resistant to previous immunosuppressive therapy. Several characteristics for KX treatment of IgAN were found in this study. First, the effect on reduction of proteinuria for IgAN is impressive. At the first follow-up, 53 (96%) patients showed a reduction in proteinuria. Thirty-four (61.8%) achieved complete/partial remission, 25 (45.5%) achieved complete remission, and 41 (74.5%) showed urinary protein excretion decreased by at least 50% from baseline. Second, proteinuria decreased rapidly after treatment. Among the 27 patients followed within 5 weeks after KX treatment, 9 (33.3%) of them achieved complete/partial remission. Third, the proteinuria could be increased rapidly within 3 months after discontinuing KX treatment. Fourth, it is interesting that women were nearly 3–4 times more likely to have partial or complete remission than men after treatment. Fifth, a dose-response effect was observed in this study, such that a higher dosage of KX was associated with higher complete remission (Table 2). Twenty patients were treated with TW, the second generation of TwHF preparations, before switching to KX therapy in this study. As KX contains more triptolide than TW (25 μg ± 20% per capsule vs. < 10 μg per tablet) [22], 45.2% of patients switching from TW therapy also achieved complete/partial remission during the KX treatment. It is not surprising that patients switching from TW had a lower partial or complete remission rate than patients switching from other therapies. These data also suggest that KX had a dose-response effect on reducing proteinuria in IgAN.

Ovarian failure is the most important adverse effect among premenopausal women taking TwHF preparation therapy [29]. TwHF preparation-induced ovarian insufficiency has also been confirmed in animal studies [30]. A meta-analysis showed that 17.6% of premenopausal women developed oligomenorrhea or menstrual irregularity during TwHF preparation treatment [31]. The high rate of oligomenorrhea or menstrual irregularity was also notable for KX in this study. Thus, there is a need for each woman of childbearing age to be informed of the risk of ovarian failure after being treated with TwHF preparations. The antifertility effect of TwHF preparations has also been demonstrated in animal studies [32–34]. Previous studies showed that decreased sperm motility occurred in 20.3% of males during TwHF preparation treatment [31]. Interestingly, a higher proteinuria remission rate was observed in women than men. Moreover, the development of oligomenorrhea or menstrual irregularity in women is also related to proteinuria remission.

Liver injury with significant elevation of serum transaminases could be observed during TwHF preparation treatment. The mechanism of triptolide-induced hepatotoxicity remains unknown. Mice or rats treated with a high dose of TwHF preparations expressed a slight increase in serum transaminases with insignificant liver damage. Thus, direct liver damage by these drugs may not be the only reason for their hepatotoxicity [35, 36]. The Liver injury could also be caused by impurities in the TwHF preparations. In this study, the liver injury occurred in three patients (5.5%) during KX treatment. However, only one patient occurred at the first follow-up. The levels of ALT and AST also increased slightly (3–4 U/L) from baseline to the first follow-up. These data suggest that KX has a mild effect on liver function. Liver function should be monitored regularly during the clinical use of TwHF preparations.

Another question that needs to be addressed is whether KX should be used in patients with impaired kidney function. In this study, the eGFR was stable after KX administration. Thus, KX is safe for patients with impaired kidney function. As shown in Table 2, eGFR at baseline was not associated with protein remission, suggesting that KX is also effective for patients with impaired kidney function. Similar to TW, KX is also associated with a decreased serum albumin level. As KX treatment continues, the serum albumin level will increase during the follow-up. KX treatment is also associated with increased total cholesterol, LDL, and HDL levels. An increased level of LDL could be associated with an increased risk of cardiovascular disease. Thus, the cardiovascular safety of KX treatment should be evaluated in the future.

## Conclusions

Our study showed that KX had a dose-response effect in the reduction proteinuria in patients with IgAN. As high incidence rate of amenorrhea were observed within 6 months of KX treatment, woman of childbearing age should to be informed of the risk of ovarian failure after being treated with TwHF preparations. The long-term safety and efficacy of KX in the treatment of IgA nephropathy should be further explored.

## Data Availability

The datasets used and analyzed during this study are available from the corresponding author on reasonable request.

## Abbreviations

IgAN: IgA nephropathy
KX: KunXian capsule
eGFR: Estimated glomerular filtration rate
TW: Tripterygium Wilfordii multiglycoside
TwHF: Tripterygium Wilfordii Hook F.
